# A cannabis oracle? Delphi method not a substitute for randomized controlled trials of cannabinoids as therapeutics

**DOI:** 10.1186/s42238-021-00074-0

**Published:** 2021-07-02

**Authors:** Kevin P. Hill, Donald I. Abrams

**Affiliations:** 1 Division of Addiction Psychiatry, Beth Israel Deaconess Medical Center, Grzymish 133, 330 Brookline Avenue, Boston, MA 02215 USA; 2 Department of Psychiatry, Harvard Medical School, Boston, USA; 3 Department of Medicine, University of California, San Francisco, USA

**Keywords:** Medical cannabis, Chronic pain, Cannabidiol, CBD, Tetrahydrocannabinol, THC, Delphi process

## Abstract

**Background:**

With millions of people using cannabinoids to treat a host of medical conditions, clinicians want guidance on how to utilize cannabinoids as pharmacotherapy in their practices. The Delphi method is a systematic, interactive forecasting method that aims to develop consensus best practices where guidelines are not available.

**Body:**

A multidisciplinary group of global cannabinoid experts utilized a modified Delphi process to develop three protocols for the dosing and administration of cannabinoids to treat chronic pain. Two protocols recommend cannabidiol (CBD), for which there is limited evidence as an analgesic, starting well below doses required for other indications. Guidance on prescribing CBD for pain may demonstrate consensus recommendations based upon suboptimal evidence.

**Conclusion:**

Consensus processes like the Delphi method are well-meaning, but they are not a substitute for rigorous RCTs with large sample sizes, adequate duration, and standardized outcome measures.

## Background

The rate and scale of cannabinoid science has not been able to keep pace with the intense clinical interest in cannabinoids. With millions of people using cannabinoids to treat myriad medical conditions, clinicians are clamoring for guidance on how to utilize cannabinoids as pharmacotherapy in their practices. Recent data suggest that three-quarters of medical trainees want more cannabis education than they currently receive (St. Pierre et al. 2020). The use of cannabis and related compounds as medicine remains a controversial topic; thus, limited clinical practice guidelines exist.

The Delphi method is a structured communication technique that was developed as a systematic, interactive forecasting method. A panel of experts answers questionnaires in two or more rounds. Anonymized summaries of responses are provided to the experts after each round and the experts are encouraged to revise their answers in light of the others’ replies. The range of answers converges toward consensus and the process is stopped after a predefined stop criterion is met.

## Main text

In this issue of *Journal of Cannabis Research*, a multidisciplinary group of global experts in the field of cannabinoids utilized a modified Delphi process to develop three protocols for the dosing and administration of cannabinoids to treat chronic pain (Bhaskar et al. 2021). The protocols—routine, conservative, and rapid—were designed based upon the desired time to achieve clinical effects. In the routine and conservative protocols, CBD-predominant cannabinoid products are started at a dose of CBD 5 mg twice daily and titrated up to CBD 40 mg daily, at which time small doses of THC are started. In the rapid protocol, patients are started on cannabinoids with THC and CBD in a 1:1 ratio at 2.5–5 mg of each cannabinoid one to two times daily and titrated to a maximum dose of THC 40 mg daily.

Guidance on prescribing CBD for pain illustrates consensus recommendations based upon suboptimal evidence. Despite hypothetical and preclinical rationales for CBD as an analgesic (Pisanti et al. 2017), limited clinical evidence, in the form of data from randomized clinical trials (RCTs), exists. Most RCT data for cannabinoids in pain is for THC alone or products with THC and CBD, such as nabiximols, or whole-plant cannabis. CBD alone has been shown to be effective in patients with pain from neurologic injuries, kidney transplantation, neuropathy, and fibromyalgia (Wade et al. 2003; Cunetti et al. 2018; Xu et al. 2019; Van De Donk et al. 2019). However, these studies utilized small sample sizes and disparate outcome measures. Similar trials of CBD for pain from Crohn’s disease and generalized chronic pain did not show benefit (Naftali et al. 2014; Notcutt et al. 2004, Cunetti et al. 2018; Capano et al. 2020), however, leaving a clear need for larger, rigorously designed trials of CBD for chronic pain.

Bhaskar and colleagues attempt to expand the limited evidence for CBD as an analgesic even further by providing dosing recommendations for CBD without presenting additional data. Starting at CBD 5 mg twice daily and titrating to 40 mg daily, while unlikely to lead to side effects, is also quite a bit lower than CBD dosing used, albeit for a variety of indications, in other clinical trials. Several rigorously designed RCTs utilized CBD doses in the range of several hundred to over a thousand milligrams. Why CBD was chosen as the initial intervention when the existing data demonstrates analgesic effects from THC-based interventions seems puzzling. Despite the input from the Delphi experts, at the end of the day, the clinician may be better off deciding which cannabinoid he or she hopes will provide analgesia and then dosing that cannabinoid appropriately by assessing the patient’s response.

## Conclusion

There is a clear need for clinical guidance on the use of cannabinoids as pharmacotherapy for chronic pain. Consensus processes like the Delphi method are well-meaning, but they are not a substitute for rigorous RCTs with large sample sizes, adequate duration, and standardized outcome measures. All key stakeholders must share the goal of using RCTs to determine cannabinoid efficacy in chronic pain, and those who can contribute to this goal should do so. We need the best science we can produce.

## Data Availability

Not applicable.

## Abbreviations

CBD: Cannabidiol
THC: Tetrahydrocannabinol

## References

[CR1] Bhaskar A, Bell A, Boivin M, et al. Consensus recommendations on dosing and administration of medical cannabis to treat chronic pain: results of a modified Delphi process. J Cannabis Res. 10.1186/s42238-021-00073-1 10.1186/s42238-021-00073-1 PMC8252988 34215346

[CR2] Capano A, Weaver R, Burkman E (2020). Evaluation of the effects of CBD hemp extract on opioid use and quality of life indicators in chronic pain patients: a prospective cohort study. Postgrad Med.

[CR3] Cunetti L, Manzo L, Peyraube R, Arnaiz J, Curi L, Orihuela S (2018). Chronic pain treatment with cannabidiol in kidney transplant patients in Uruguay. Transplant Proc.

[CR4] Naftali T, Mechulam R, Lev LB, Konikoff FM (2014). Cannabis for inflammatory bowel disease. Dig Dis.

[CR5] Notcutt W, Price M, Miller R (2004). Initial experiences with medicinal extracts of cannabis for chronic pain: results from 34 ‘N of 1’ studies. Anaesthesia.

[CR6] Pisanti S, Malfitano AM, Ciaglia E (2017). Cannabidiol: state of the art and new challenges for therapeutic applications. Pharmacol Ther.

[CR7] St. Pierre M, Matthews L, Walsh Z (2020). Cannabis education needs assessment among Canadian physicians-in-training. Complement Ther Med.

[CR8] Van De Donk T, Niesters M, Kowal MA, Olofsen E, Dahan A, Van Velzen M (2019). An experimental randomized study on the analgesic effects of pharmaceutical-grade cannabis in chronic pain patients with fibromyalgia. Pain.

[CR9] Wade DT, Robson P, House H, Makela P, Aram J (2003). A preliminary controlled study to determine whether whole-plant cannabis extracts can improve intractable neurogenic symptoms. Clin Rehabil.

[CR10] Xu DH, Cullen BD, Tang M, Fang Y (2019). The effectiveness of topical cannabidiol oil in symptomatic relief of peripheral neuropathy of the lower extremities. Curr Pharm Biotechnol.

